# Modeling the enablers of online consumer engagement and platform preference in online food delivery platforms during COVID-19

**DOI:** 10.1186/s43093-022-00119-7

**Published:** 2022-04-13

**Authors:** Aqib Habib, Muhammad Irfan, Mohsin Shahzad

**Affiliations:** 1 School of Economics and Management, Dalian University of Technology, Dalian, 116024 People’s Republic of China; 2 Division of Management & Administrative Sciences, University of Education, Lower Mall Campus, Lahore, 54,000 Pakistan

**Keywords:** Food delivery apps, Platform preference, Online consumer engagement, PLS-SEM

## Abstract

Due to the COVID-19 outbreak globally during 2020, the usage and virtues of food delivery apps (FDA) have increased immensely, facilitating the consumer to access the food and food providers to keep functioning. However, this study aims to investigate the enablers of online consumer engagement (OCE) and platform preference in the foodservice industry, keeping in view the moderating role of peer pressure by following uses and gratifications theory (UGT). The data were collected from 322 FDA's user in China during the COVID-19 lockdown and analyzed employing partial least-square structural equation modeling (PLS-SEM). PLS-SEM results revealed that consumer’s self-concept and platform interactivity affect OCE and platform preference. Further, OCE mediates the effect of the relationship between platform interactivity, self-concept, and platform preference. Furthermore, peer pressure significantly moderates the relationship between OCE and platform preference. This research contributes to the prevailing body of literature in a novel way by employing UGT on consumer behavior in the FDA. The study has value for online food businesses and implications for consumers, retailers, and practitioners to formulate and implement value-added strategies in a consumption-oriented emerging economy.

## Introduction

Globalization is continuously persuading the ways in which consumers interact with different brands online to offline (O2O). It is a kind of e-commerce in which shoppers attracted a service and product online and actuated to complete the deal offline [1]. This trend has been accelerated by the exponential growth of the use of online food delivery applications (FDAs), social networking sites (SNSs), smartphone devices, and information and communication technologies (ICT) due to their real-time connectivity and accessibility, which influenced online food delivery business a prominent place among busy diners preferring speed and convenience [1]. According to a study from the Global Association of Mobile Operators, worldwide mobile phone users surpassed 5.1 billion in the current year; over 1.2 billion users are accounted for in China [2]. These advanced technology and analytics have driven market competition and improved customer interactions [3]. This is imperative for businesses to recognize consumers' behavioral trends to keep them engaged. FDA is an innovative way to buy food [4]. As FDA (online-to-offline) mobile services are gaining popularity, consumers’ expectations about service delivery have also immensely augmented [3]. The online food industry is recognized to be a rapidly growing industry with approximate revenues of the US $137.6 billion by 2023 [5].

The FDA's revenue in major countries shows remarkable growth in the COVID-19 outbreak, and China is leading from the front end with forecast revenue being approximately 51,514 million USD with leading platform Meituan and Eleme following the USA with 26,527 million USD in 2020. Globally, FDA is getting to be progressively well-acknowledged and grasp by young grownups, and this trend is drift more apparent in China [1]. It is the source of employment as these FDA platforms such as Meituan and Eleme employ approximately 1.7 million workers for deliveries in China [6]. These statistics provided evidence that during the lockdown by COVID-19, the FDA being accredited for empowering many food traders to survive. Accordingly, factors motivating users to use FDA incessantly under the COVID-19 epidemic condition are indispensable for pertinent stakeholders to understand customers’ expectations and requirement [7].

Subsequently, how to better engage customers through innovative and smart media has become a core challenge for researchers and practitioners to recognize and leverage online customer interaction in the online food industry. Social media's proliferation has transformed the marketer–customer experience, allowing consumers to engage directly through real-time interactions with the organization [8, 9]. Recent statistics indicate similar trends, 25 percent of installed mobile apps were never used, and 26 percent of installed mobile apps were discontinued after first use [10]. As consumers in the foodservice industry are highly fickle, the industry needs to keep it up to date with variations in taste, trend, and accessibility. FDA provides effective means to approach potential customers and deliver customized value-added services. Annie [11] found that Chinese citizens install nearly 40 apps monthly, exceeding other nations' statistics, such as France, the USA, and India. FDA is one of the fastest growing e-commerce apps on different app stores besides entertainment, gaming, and social commerce apps [12].

With the continuous evolution of mobile internet, the foodservice industry relies on social media technology as a key information and marketing tool [13]. Strong interactive relationships among customers and firms help to meet one or more essential self-defined needs. Such identification empowers customers to benefit from the desired level of online consumer engagement (OCE) [14]. Existing literature identified that OCE plays a vital role in creating exciting customer experiences [15]. Thus, it is imperative to understand customer interactions to develop stronger emotional ties with online market players [16]. In services marketing research, consumer engagement in online platforms is a key explanatory factor of platform choice [17]. Previous studies revealed that social networking platforms’ upsurge, mainly social media brand communities, positively influences brand engagement and platform preference [18]. Jahn and Kunz [19] described that self-concept is a prominent element that influences the OCE, which helps to attain customer satisfaction through emotional affection. The notion of self-concept is divided into perceived value, perceived quality, and self-brand image congruency and is antecedents of consumer engagement behaviors [16].

On the other hand, peer pressure is also a prominent factor influencing users to create profiles, exchange information, connect, and interact on a particular platform with other users [20]. FDA usage strongly impacts friends' social relationships due to peer pressure [1]. Extant literature identified that increased competition in the online food service industry was key in identifying factors that engage consumers in purchasing while influencing their channel choice behavior and open new prospects for researchers in the current arena [7]. Further, the preceding researchers placed platform interactivity as a vital means to engage consumers because brands are increasingly investing in programs to facilitate retailer–consumer connections [21].

Despite having a lot of literature on social commerce, the aspects and antecedents of platform preference with OCE's mediating role in the online food delivery industry are still not appropriately focused. Therefore, this research investigates how platform interactivity and self-concept affect OCE in an encompassing model that further affects platform preference directly and with the moderating effect of peer pressure. Therefore, the problems mentioned above and the literature gap compelled this examination following the research questions and intended to diminish the uncertain situation surrounding these associations.

RQ1:How does platform interactivity and self-concept effect OCE?

RQ1a: How does OCE mediate the relationship among platform interactivity, self-concept, and platform preference?

RQ2:How does peer pressure moderate the relationship between OCE and platform preference?

To evaluate the hypotheses, data were collected from FDA users from China during the COVID-19 lockdown. By responding to the above-mentioned research questions, the current study contributes to the prior literature. First, this research fills the gap by evaluating the relationship among platform interactivity, self-concept, OCE, and platform preference in an encompassing model using uses and gratification theory (UGT) through structural equation modeling (SEM). Second, this study's findings empower the experts and marketers to integrate various strategies in developing consumer engagement and platform preferences, thus encouraging potential customers to choose relevant FDA. Third, this study engrossed in the different underexplored OCE antecedents alongside OCE mediation and peer pressure moderation.

## Theoretical background and literature review

### Uses and gratification theory (UGT)

Uses and gratifications theory (UGT) evolves from motivation and needs theories [22]. It allows scholars to recognize how and why customers are deliberately searching for choosing a particular application to meet their needs [23]. UGT is used to find the motives behind users’ choices for selecting a particular application to fulfill their needs. From the 1980s onward, the UGT has become a common theoretical framework for understanding the use of various media forms [22].

Researchers assess OCE in the digital media by the number of likes, comments, the volume of reviews, and shares. When consumers interact with brands or brand-generated content, these interactions are conceptualized theoretically as OCE, which leads a consumer to select the platform preference [24]. With the expansion of social media, researchers protracted the choice of UGT to theorize the motivations behind the antecedents of OCE [25]. Following UGT, past studies demonstrated that it has been used for the application of technology, including the use of the internet and online community networking, and is an extension to the technology acceptance model (TAM) and theory of reasoned action (TRA) [26]. It helps to understand the reasons for the excessive use of social media by adolescents and the consequences of social media adoption [27]. UGT expresses the gratification level of mobile SNS use and its effect on users' behavior [28]. It further describes individuals attempting to accomplish their leisure and informational needs [29]. Ray et al. [25] determined the factors that influence the acceptance of FDAs following UGT. Researchers recently adopted UGT to investigate the purchase intention and intensive social media usage to gratify themselves [30, 31]. Furthermore, Busalim et al. [32] identified critical factors that affect consumer engagement in social commerce following UGT. Thus, this study researcher adopted UGT to bridge the prevailing literature gap by identifying the users’ motivation underlying FDA usage. Further, the literature overview is provided in Table 1, indicating that prior researchers adopted UGT in similar research areas.

**Table 1:** Detailed literature review

Authors	Methods and theory	Objectives	Integrated constructs
Busalim et al*.*[32]	203 Survey UGT	To identify factors affecting consumer engagement in social commerce	Customer engagement behavior, social interaction, technological factors, interactivity, and motivational factors
Troise et al*.*[6]	425 Survey TPB	To address the gap on the antecedents behind the consumer’s choice to adopt online food delivery apps	Attitude, Perceived usefulness, Perceived behavior control, Subjective norms, Perceived usefulness, Convenience, Various food choices, Perceived ease of use
Zhao and Bacao [7]	532 Survey UGT	To fill the gap between factors that determines the intention of users to use food delivery applications during COVID-19	Continuance intention, Trust, Social influence, Effort expectancy, Satisfaction, Performance expectancy
Li and Mirosa [1]	Review Studies	To identify all stakeholders’ opportunities for intervention, particularly online FDAs, business experts, customers and academics, policymakers, to optimize its positive impact and reduce its adverse effects	Review Studies
Kaur et al*.*[31]	309 Survey UGT	To investigate both the virtual goods purchase intention on continuance intention and mobile instant messaging (MIM) toward MIM itself	Escape, Exposure, Affection, Entertainment, Purchase Intention, Continuation Intentions, Information seeking, Social sharing
Raza et al*.*[30]	250 Survey UGT and TPB	To explore the intense use of Facebook among the students by incorporating the structure of UGT	Escape and Intensive, Ease of use and Intensive, Information seeking and Intensive, Subjective norm and Intensive, Social relationship, Education, Career opportunities, Perceived behavioral control, attitude
Ray et al*.*[25]	395 Survey UGT	To evaluate the variables affecting the endorsement of the FDAs	Delivery Experience, Ease of use, listing, Customer Experience, convenience, Quality control, Search of restaurants, Societal Pressure,
Lim and Kumar [33]	796 Survey UGT	To recognize the motives of customers to engage in online social networking	Information, Incentives, Entertainment, Connectedness, Brand likability, Brand attachment, Global and local businesses, Age, Gender, brand online social networking commitment
Gan and Li [34]	297 Survey UGT	This study sheds light on reinforcing the functions of various gratifications in influencing consumers' continuing actions of information systems in the social network	Social gratification, Technology gratification, Utilitarian gratification, continuance intention, hedonic gratification
Heravi, Mubarak and Raymond Choo [35]	521 Survey UGT	To investigate privacy attitudes and behaviors in online social networks (OSNs)	Information privacy in OSN, Motives and information privacy, Self-disclosure, Information privacy, and self-disclosure, Privacy behavior
Phua, Jin and Kim [29]	305 Survey UGT	Compared social capital bridging and bonding between regular Facebook, Twitter, Instagram, and Snapchat users and different intervening variables affecting their relationship	Introversion, SNS privacy concerns, SNS trust, tie strength, and homophile, SNS intensity, Bridging and bonding social capital, social networking sites use
Punyanunt-Carter, De La Cruz and Wrench [36]	475 Survey UGT	To analyze the desired uses and rewards obtained via Snapchat, especially within the context of gender	Life Orientation, Communication Apprehension, Snapchat Satisfaction, Communication Apprehension, Snapchat Intensity, Communication Motives, Needs Satisfaction, Exhibitionism
Malik, Dhir and Nieminen [37]	368 Survey UGT	To determine the various gratifications behind Facebook-based photograph sharing related activities	Information sharing, Social interaction, Entertainment, Disclosure, Affection seeking, Habitual pastime, Social influence, Attention seeking,
Ifinedo [38]	797 Survey UGT	To explore the growing use of SNSs	Uses and gratification theory, Pervasive adoption, Social influence, individualism and collectivism, behavioral intention to use SNSs,
Ha et al*.*[28]	641 Survey UGT	To identify the gratifications of mobile SNS usage and their impact on user behavior	Cognitive, integrative, social integrative, mobile convenience, hedonic, Actual use, attitude toward mobile SNS

Theory of planned behavior (TPB); uses and gratifications theory (UGT); unified theory of use and acceptance of technology (UTAUT)

### Platform preference (PFP)

In marketing and other related research areas, “platform preference” is a well-established concept [39]. It is defined as “the setting by an individual of one thing before or above another thing because of a notion of betterness”[40]. When it comes to food sector customers, share different tastes and experiences. They may also have diverse expectations about the services and quality of particular FDA and may also have various opinions about their perceived value levels for using those FDA [4]. Accordingly, the niche of food delivery services using mobile phone apps has developed a swiftly growing phenomenon among Chinese take-out eateries as an appropriate way for these companies to upsurge sales revenue [41]. FDA and delivery men providing a critical lifeline during the epidemic (COVID-19) for the millions of people quarantined at different places. The FDA provided food and allowed people who prepared or delivered the food [42]. Eateries can engage in crowdsourcing logistics, a network of diverse delivery men who are independent suppliers, a model that offers an efficient, low-cost way to deliver food [43]. Previous researchers identified that consumer satisfaction significantly affects platform preference [44]. Over the past few years, the emergence of new and advanced technologies has provided new opportunities for both businesses and their customers [3]. An increasing number of mobile users in China tap their tablets or smartphones to order food on FDA, such as Meituan, Eleme, Koubei, and Baidu, which makes it possible to order food right to their doorstep from their preferred food providers with just a few quick clicks. FDA in China has been growing and now has become the eventual platform for sale revenue generation [45]. Further, consumers adopt different platforms due to marketers' different marketing strategies, such as discounts, free meals, free delivery. These FDA services are inspiring consumers to abandon cooking at home or going out to a restaurant to eat [1]. The primary research goal is to discover and investigate the factors influencing consumer platform preference grounded on their characteristics.

### Online consumer engagement (OCE)

In the current context, OCE refers to the “level of a customer’s physical, cognitive, and emotional existence in their rapport with a platform.” Brodie et al*. *[17] defined engagement as “a psychological state occurring through collaborative, co-creative, focal agent/object user interactions.” OCE meaning and views are diverse; it is typically seen as a motivational context-dependent state which has a behavior (i.e., negative or positive) and involves a subject (i.e., the consumer) and an object (e.g., brand, platform, company, channel, etc.). The individual can online engage themselves straight through the restaurant’s online platform or via a third-party platform [46]. These third-party platforms differ from country to countries, such as Eleme and Meituan, in China and Uber eats in the USA [1]. In a consumer engagement survey, 90% of companies stated that OCE is either “essential” or “important” to their organizations [47]. It has become a significant concern for online retailers. Different databases, such as “Magneto” highly engaged customers, convince their families and relatives to become new and loyal customers. OCE produces 23% more income due to consumers spending on every purchase of new product offerings [48]. It has been measured as a deliberate imperative triggered by marketers to establish and maintain a competitive edge [17]. From the perspective of this research, OCE is incorporated as a core enabler of platform preference by platform interactivity and self-concept. Further, this study also tests OCE's mediating role in the relationship between platform interactivity, self-concept, and platform preference. Businesses must optimize their marketing efforts to involve consumers, as engaged consumers are more emotional and loyal connected to the platform [49].

### Platform interactivity (PI)

Interactivity is characterized as a consumer's perception of participating in timely two-way communication with a refereed individual. Interactivity in the online platform is an essential atmospheric predictor that activates consumers’ emotional and cognitive state and, consequently, their behavioral response [50]. Hoffman DL [51] distinguished two levels of interactivity: human and machine interactivity. Human interactivity occurs between a customer and agent, while machine interactivity arises between humans and machines to access hypermedia content. Mobile app interaction is a better platform for handling user experiences than traditional ones [7]. This two-way interaction is a significant driver of brand interaction and represents its essence [52]. According to Kohler et al*.*[53], consumers engage more intensively having an online inspiring, involving, and enjoyable, interactive experience. Marketers need to create a strategy that can engage customers in the social media environment and allow them to interact freely and directly with a specific platform regardless of time, content, communication frequency, or location.

### Self-concept (SC)

Self-concept has emerged among various psychological influences as the central theme. Consumers’ perceived self-concept is a complex and significant driver of brand-related behavior, like platform preferences [54]. Sirgy et al*. *[55] identified various psychological constructs of self-concept, as it is a multidimensional point of view classified into actual self and ideal self. An actual self is identified that how a customer sees himself, though the ideal self is identified that how a client might want to see oneself [56]. The concept is established on the fundamental theory of self‐schemas by Markus [57]. It can affect judgments and decisions; further, it can also undoubtedly influence consumer behavior [57].

Moreover, the advanced levels of brand engagement in self-concept are associated with preferences and purchase intentions [58]. Research has shown that consumers buy products congruent with or enhance their self-concept [46]. Self-concept is a useful psychological factor of consumer decision making. For the current study, self-concept is divided into perceived value, perceived quality, and self-brand image congruency; these are antecedents of consumer engagement behaviors [59]. Many notable previous studies have also taken either all these dimensions or two of them under the umbrella of self-concept [60]. However, for this research, we have adopted perceived value, perceived quality, and self-brand image congruency for accumulating self-concept. Perceived quality is a result of consumers’ subjective judgment on a product [60]. Perceived value and perceived quality are a strong determinant of post-purchase attitudes, intentions, and behavior [55]. These are consumer judgments on the accumulative product assistances and a subjective feeling on product quality [61].

Further, Byun et al*.*[62] determined that self-brand image congruency influences customers’ self-concept and product image. Customers have a positive feeling about a particular brand when satisfied with that brand [63]. Actual self-image and product image influence customers’ attitudes and behavior [61]. In spite of the increasing appreciation of the importance of perceived value, quality, and brand-image congruency, only a few studies have investigated how brand experience and perceived healthy function in the food industry. Customers tend to take the brand as part of their self-concept [64]. Following these arguments, it is proposed that the engagement toward the brand that supporters create from the anticipated enticing effect of the influencers will influence in terms of a more noteworthy expectation to incline toward the platform and purchase decisions.

### Peer pressure (PP)

Peer pressure means that the individual decisions are inclined by their social networks, as individuals often consider others’ views when determining whether or not to use a given platform [65]. Venkatesh et al*. *[66] abstracted peer pressure as “the extent to which an individual perceives that important others believe he or she should apply the new system.” Peer pressure is also considered social pressure. It has been among the most significant factors measured about consumers using or refusing mobile commerce applications [67]. Eke and Singhry [68] found that peer pressure has a role in expecting the consumer’s intention to use mobile commerce apps. Dhir et al*. *[69] found that social influence positively affects the choice to use mobile payment. In the context of the current study, the impact of peer pressure on customer engagement can only be fully exploited by the web users’ willingness to share and interconnect information and their views on specific services and products. Based on consumer socialization theory, the mechanism by which distinct consumers learn skills, information and attitude from others through contact, which then assists them as consumers on the marketplace, it may be argued that communication among consumers affects the cognition, affection, and actions of each other [70].

## Research model and hypotheses development

**Fig. 1:**
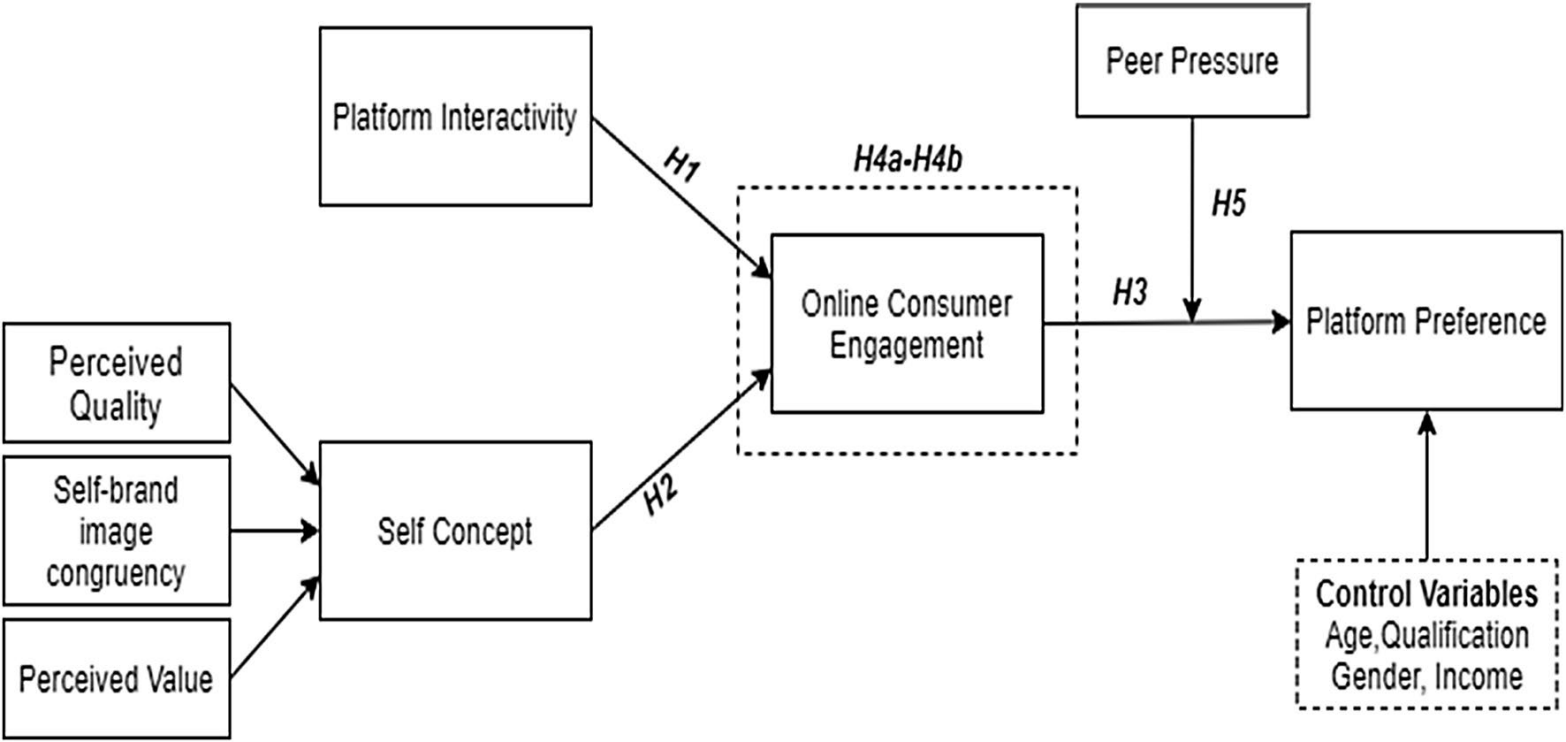
Research model of the study

The research model in Fig. 1 depicts the influence of platform interactivity and self-concept on OCE and further on platform preference. Further, it illustrates OCE's meditating role among platform interactivity, self-concept, and platform preference following the UGT. Moreover, the moderating role of peer pressure is also examined among these relationships.

### Relationship between platform interactivity and OCE

Interactivity is characterized as the consumer’s perception of two-way communication promptly using a mediating channel. Platform interactivity is a significant predictor that activates consumers’ emotional and cognitive state and, consequently, their behavioral reaction [50]. Mobile apps are useful media for handling user experiences than traditional media. This two-way interaction is a significant driver of brand engagement [52].

Kohler et al*.*[53] consumers engage more intensively while having an online inspiring, involving, and enjoyable, interactive experience. Marketers need to create a strategy that can engage customers in the social media environment and interact freely on a specific platform regardless of time, content, communication frequency, or location.

The researcher identified platform interactivity as a prime precursor of brand engagement [71]. Islam and Rahman [72] defined interactivity as positively impacting customer interaction within an online brand environment, indicating that interactivity plays an essential role in concerning customers with brands. Interactivity and value co-creation are realized based on consumer engagement [17]. Further, platform interactivity gives ease of communication, participation, and collaboration between customers and firms in the value creation process [73]. Firms have various means to manage customer relationships through marketing channels by offering value-added features such as customization, interactivity, and design that enable customers to engage the firm in two-way communication while giving a unique marketing experience [74]. Consumer desire to tailor their experience on the platform is a vital part of customer engagement [75].

Further, it can improve an organization's telepresence, influencing consumer engagement (e.g., efficient cognitive processing, instrumental, and experiential value). Mobile apps' collaborative features allow retailers to offer unique user experience and increase customer engagement [76]. Therefore, the following hypothesis was proposed:

#### H1

Platform interactivity has a positive impact on online consumer engagement.

### Relationship between self-concept and OCE

The perceived self-concept is a complicated driver of brand-conscious behavior, like platform preferences [54]. The higher levels of self-concept and brand engagement are correlated with preferences or buying intentions. Following, it is implied that the engagement to the brand that supporters build from the influencers' anticipated convincing effect would influence the terms of a greater desire to prefer the platform and purchase. Additionally, consumers often generate and express their own brand-related content on social media, influencing brand perceptions, and behaviors. Byun et al*. *[62] determined that self-brand image congruency influences congruence between customers’ self-concept and product image. The researcher identified that customers have a positive and strong feeling on a particular brand when they feel consistency with such a brand [63]. The congruence influences emotional attachment among customers’ self-concept and brand image [56]. Accordingly, understanding the mechanism of service brand-related attributes in the formation of self-concept behavior is meant for service providers and marketers because it provides guidelines for developing the most effective advertising and promotion strategies. Customers tend to adopt the brand as part of their self-concept [64].

Self-concept is a general tendency of a customer to associate a brand or platform with his buying portfolio. The extent to which customers experience a brand is consistent with their self-concept and is a driver of consumer engagement. Brand image should reflect consumer personality attributes and reflect consumer engagement behavior [77]. Consumer engagement is affected by the consumer’s self-image and brand image [56]. An essential distinction exists among engagement, self-concept, and the associated branding frameworks, such as self-brand [78]. Once the consumer’s self-concept goal is attained, they tend to build an emotional affection toward a brand [63]. The more a platform appealing to customers’ self-concept, the stronger is the level of their association to product buying on that platform [64]. Astakhova et al*. *[56] stated that "ideal social self-image" is a key factor and part of self-concept that affects brand engagement. Hence, we proposed the following hypothesis:

#### H2

Self-concept has a positive impact on online consumer engagement.

### Relationship between OCE and platform preference

OCE is realized as a predictor of platform preference, which leads to increase purchase intention [79]. The firm can interact, offer promotions, conduct surveys, post-valuable content, communicate, engage, and build better connections with customers for value addition [80]. The ability to engage customers is significant for capturing and maintaining market dominance in extremely competitive environments, especially in the mobile app market, as the number of mobile apps is rapidly growing [3]. One possible explanation for this observed trend is that customers are flooded continuously by rising numbers of mobile apps. How consumers engage with platforms and not switch to other platforms after adopting the specific application remains under investigation.

Traditionally preference is defined as "the placement of one thing before or above another by an individual on account of a belief of bitterness”[40]. When it comes to food, consumers may have diverse perceptions about the quality and facilities of particular food delivery applications and may also have different views regarding their perceived value levels for using such FDA [6]. FDA functions within online food delivery as they empower food ordering through different apps online [81]. The emerging technologies have provided opportunities for both businesses and customers to get value for their investment. The research findings reveal that interactivity with applications causes customer engagement, ultimately influencing platform choice behavior [82]. Hence the following hypothesis can be proposed:

#### H3

Online consumer engagement has a positive impact on platform preference.

### The mediating role of OCE

Engagement is described as a psychological condition occurring through collaborative, co-creative, focal agent/object user interactions [17]. Though definition and opinions of the term differ, customer engagement is typically seen as an information-dependent psychological state [83], which has a positive or negative behavior [16] that includes a subject (i.e., the consumer) and an object (e.g., company, brand, platform, channel, etc.).

Customer engagement has been considered a deliberate necessity for marketers to create and retain a competitive advantage over others [84]. Businesses should optimize their marketing strategies to involve customers, as engaged customers are more committed and loyal to the platform or channel [48]. There is a profound relationship between platform interactivity, OCE, and platform preference. Firms proceed with a customer-centric approach and invest in improving consumer engagement through virtual interaction [85]. Retailers’ interactions through social networking sites are supposed to create long-lasting engagement that can lead to a psychological and prolonged relationship between both parties. Therefore, engaged customers are likely to favor a particular platform because they are satisfied and committed and have an emotional attachment to the brand.

Considering the online context, engagement is a significant and cognitive connection to dynamic interaction with the manufacturer as embodied by the platform or other computer-mediated sources designed to promote product interest [86]. In effect, consumers interact to a retailer's application and experience cognitive telepresence or cognitive engagement in the platform. In this way, consistent cognitive processing meets the interpersonal (usefulness and relevance) and consumer's empirical value. Thus, the following hypotheses were proposed.

#### H4a

Online consumer engagement significantly mediates the relationship between platform interactivity and platform preference.

#### H4b

Online consumer engagement significantly mediates the relationship between self-concept and platform preference.

## Moderating role of peer pressure

Peer pressure refers to the degree to which individuals’ decisions are influenced by their social networks, as individuals often consider others’ views when determining whether or not to use a given platform [87]. The internet users’ ability to exchange and convey information and their opinions on other products and services will completely leverage the social network's impact on customer engagement. Based on consumer socialization theory, consumers’ interaction influences each other's affection, cognition, and behavior [88]. Consumers thus acquire attitudes and behaviors related to consumption by learning from socialization practitioners by watching or communicating with them. Associated with the current study, peer pressure has been acknowledged as a significant decisive users’ intention to use an online-to-offline delivery service [6]. These day’s businesses need to use the power of social media not only to communicate about their goods and brand promotions but to engage customers in an experiential environment [84]. Social networks, peer power, and community elements are critical because of the social connections between individuals, sellers, and buyer groups [89]. Community influence or peer pressure has been identified as a critical driver of consumer engagement in an online platform [90]. In effect, the user's content (referrals, comments, ratings, or testimonials) contributes to affective, cognitive, and behavioral responses. Social commerce platforms work as recommending systems and provide real-time opportunities to spread consumer reviews, assessments, and product recommendations, which subsequently affect other consumers' trust in retailers or that channel [91]. In a nutshell, peer pressure is a key factor in customers adopting or rejecting a social commerce industry channel.

### H5

Peer pressure significantly moderates the relationship between OCE and platform preference.

## Methods

### Sample and data collection

The current study is focused on meal order online food in China by using different FDA. The active users of Eleme, Meituan, Baidu, and Koubei were brought under this study's scope. Social media has been combined as the most favored platform for information appropriate to hotels and restaurants [83]. A survey method was adopted for data collection. For this purpose, an online questionnaire was designed to evaluate the hypotheses [92]. This unit of analysis for our study was user of FDA because this study aimed to measure OCE and platform preferences in online food delivery industry. We adopted simple random sampling to collect data from target population because it is convenient and saves time and resources when population is scattered and large in number. The questionnaire was first developed in the English language; however, it was translated into the Chinese language, completed with three bilingual experts’ help to ascertain and ensure the content quality [93]. First of all, we started spreading our questionnaires in different social media groups inside mainland China. We used different means to approach potential respondents through Weibo, WeChat, phone calls, and email to deliver questionnaires, followed by reminder emails and telephone calls from March to April 2020 to maximize our research validation. Weibo and WeChat are widely used means of communication across mainland China. WeChat pay is actively used by users of various FDA to pay online for food shopping. We also delivered questionnaire in those community groups specific for online food shopping. For this study, we targeted 600 respondents from different regions of China who use the FDA. A set of 359 completed questionnaires was returned. Thirty-seven questionnaires were removed because of judgmental errors as the required respondents did not fill these responses.

A total of 322 responses were completed giving a response rate of 53.66%, which is adequate in survey studies [94]. Almost 71% of the respondents were Chinese nationals. The age of the majority of the respondents (30%) was between 31 and 35 years. Details of demographic attributes are given in Table 2. For sample size, this study espoused the ten times rule, which Hair Jr et al. [95] endorsed, that is, “10 times the largest number of structural paths directed at a particular latent construct in a structural model.”

**Table 2:** Demographic details of respondents (n = 322)

Attributes	Characteristics	Frequency	%
Gender	Male	176	54.65
	Female	135	41.92
	Prefer not to say	11	3.41
Age	20–25	74	22.98
	26–30	83	25.77
	31–35	97	30.12
	36 and above	68	21.11
Nationality	Chinese	182	56.52
	Foreigners	140	43.47
Education level	Undergraduate	85	26.39
	Graduate	139	43.16
	Post-graduate	98	30.43
FDA usage Frequency	Minimal	81	25.15
	High	241	74.84

The potential for non-response bias was checked by observing the Chi-square of early and late respondents’ responses by choosing the first 20 percent and the last 20 percent of respondents. The outcomes indicated no significant difference between responses of early and late respondents on key measures.

## Measures

With the help of prior research literature, an online survey was developed to measure the hypothesized constructs. This study's major constructs consist of self-concept, platform interactivity, online consumer engagement, peer pressure, and platform preference. We used 7-point Likert scales to measure the construct items, ranging from "strongly disagree" to "strongly agree." In the beginning of our survey, we mentioned that only respondents who are buying food online can proceed further. A pilot study was conducted to ensure content validity from 50 respondents sample. Self-concept was measured using the 12 items distributing these into three dimensions: perceived quality and perceived value, each containing four items adapted from Dwivedi [96] and self-image congruence by four items from Jiseon [54]. Further, platform interactivity encompassing five items was adapted from Etemad-Sajadi [97]. Online consumer engagement was measured by Jiménez-Castillo and Sánchez-Fernández [98] containing four items. Similarly, peer pressure containing four items was adapted from Jiménez-Castillo and Sánchez-Fernández [98]. Lastly, platform preference was adopted from Johnson, Herrmann, and Huber [99], containing seven items.

## Results

We analyzed the data by using SmartPLS version 3.2.8 and IBM SPSS version 24, using partial least-squares structural equation modeling (PLS-SEM). This method is most endorsed when the study focuses on predicting and exploring the exogenous variables. It can cater both the measurement and structural model concurrently. So, PLS-SEM is the best prediction-oriented method and seems appropriate for this study [95].

### Common method variance (CMB)

We performed Harman single-factor test employing principal component analysis by varimax rotation to test CMB's existence. The maximum variance explained by a first factor was 34.05%, which is lower than the 40%, demonstrating that CMB was not an issue [95]. Secondly, following Kock [100], variance inflation factor (VIF) values were assessed. All the values were below the threshold value of 3.3, signifying that the model does not have any CMB issue [100].

### Measurement model analysis

Before examining the hypothesized relations, the quality of the measurement model was measured by various means. First, we checked the normality of data through the KMO test of sampling adequacy and Bartlett test of sphericity using SPSS. The KMO value of 0.943 approximates Chi-squares (5626.64) and Bartlett’s test degree of freedom 496; *p* < 0.001 showed that our sample is normally distributed and suitable for regression analysis [101]. Likewise, SC is a second-order formative construct, and the conventional approach is not appropriate to assess their reliability and validity. Therefore, following Petter et al. [102] recommendation, outer weights of first-order constructs for SC are shown in Table 4, which shows evidence for construct validity [102].

#### Convergent validity

Table 3 gives a detailed description of the quality of the measurement model by presenting the values of factor loadings, composite reliability (CR), average variance extracted (AVE), and Cronbach’s alpha (Alpha) to measure convergent validity [103]. The confirmatory factor analysis (CFA) results reveal that all item's factor loadings were more significant than 0.50. Similarly, the average variance-extracted (AVE) values were more significant than the minimum benchmark of 0.50. Moreover, all constructs CA and CR values were within the recommended range, giving assurance of convergent validity and reliability [104].

**Table 3:** Quality of measurement model

Constructs	Items	Loadings	CA	CR	AVE
Platform interactivity (PI)	PI1	0.86	0.90	0.90	0.69
	PI2	0.98			
	PI3	0.814			
	PI4	0.79			
	PI5	0.71			
Perceived quality (PQ)	PQ1	0.84	0.91	0.93	0.74
	PQ2	0.86			
	PQ3	0.82			
	PQ4	0.89			
Self-brand image congruency (BIC)	BIC1	0.89	0.90	0.91	0.70
	BIC2	0.79			
	BIC3	0.88			
	BIC4	0.77			
Perceived value (PV)	PV1	0.89	0.87	0.88	0.66
	PV2	0.75			
	PV3	0.84			
	PV4	0.75			
Online consumer engagement (OCE)	OCE1	0.80	0.88	0.89	0.67
	OCE2	0.82			
	OCE3	0.85			
	OCE4	0.77			
Peer pressure (PP)	PP1	0.76	0.90	0.91	0.69
	PP2	0.86			
	PP3	0.87			
	PP4	0.84			
Platform preference (PFP)	CP1	0.79	0.92	0.92	0.63
	CP2	0.80			
	CP3	0.72			
	CP4	0.80			
	CP5	0.85			
	CP6	0.83			
	CP7	0.86

**Table 4:** Assessment of formative constructs

Latent Constructs	Outer Weights	t-value	P-value
PV- > SC	0.352	34.994	0.000
PQ- > SC	0.379	30.554	0.000
BIC- > SC	0.371	30.813	0.000

*p*-values = 0.000 shows significance level ***

#### Discriminant validity

We evaluated discriminant validity by observing the outer- and inner-variance inflation factor (VIF) values. The highest outer VIF value was 3.24, while the highest inner VIF value was 1.45, which was less than the cutoff value of 5.0, demonstrating that the data have no multicollinearity issue [105]. Next, following the approach of Fornell and Larcker [106], we observed the correlation of all latent constructs and compared them with the square root of their respective average variance-extracted values in the correlation. Table 5 indicates that AVE's square root (in bold) is higher than the correlation values of other constructs in both horizontal and vertical sides, which shows no discriminant validity issues. Further, we estimated the Heterotrait–Monotrait (HTMT) ratio. The results (Table 6) suggested that all HTMT values were below the threshold level of 0.85, indicating no issue of multicollinearity [104, 105].

**Table 5:** Discriminant validity (Fornell–Larcker criterion)

	PFP	OCE	PQ	PV	PI	PIC	PI
PFP	0.79						
OCE	0.73	0.82					
PQ	0.75	0.63	0.86				
PV	0.72	0.65	0.71	0.81			
PP	0.79	0.81	0.69	0.75	0.83		
BIC	0.77	0.75	0.75	0.75	0.79	0.84	
PI	0.64	0.72	0.69	0.72	0.71	0.65	0.83

**PFP**: Platform preference, **OCE**: online consumer engagement, **PI:** platform

interactivity, **PQ**: product quality, **PV:** perceived value, **PP**: peer pressure**,**

**BIC:** self-brand image congruency

**Table 6:** Discriminant validity (HTMT criterion)

	PFP	OCE	PQ	PV	PI	PIC	PI
PFP							
OCE	0.73						
PQ	0.76	0.63					
PV	0.76	0.65	0.76				
PP	0.79	0.84	0.69	0.75			
BIC	0.83	0.75	0.75	0.75	0.79		
PI	0.64	0.72	0.71	0.73	0.71	0.65	

#### Structural model analysis

At first, the regression analysis was applied to test the anticipated hypotheses. Table 7 summarizes the findings of regression analysis using SmartPLS. The results specify significant positive effects of both antecedents supporting H1 and H2. Also, OCE has a direct positive impact on platform preference supporting H3. Lastly, peer pressure significantly moderates the relationship of OCE and platform preference, supporting H5. The age, gender, qualification, and income were used as control variables having no significant effect.

**Table 7:** Hypotheses testing

H	Structural Paths	Path coeff	t-value	F^2^	Effect Size	Decision
H1	PI → OCE	0.35***	4.03	0.19	Small	Supported
H2	SC → OCE	0.43***	5.04	0.21	Moderate	Supported
H3	OCE → PFP	0.19*	2.29	0.139	Small	Supported
H5	PP*OCE → PFP	0.13*	2.13	0.31	Large	Supported

*P* < 0.05*, *p* < 0.001***

The results of R^2^ values indicate that OCE and platform preference had R^2^ values of 0.61 (61%) and 0.64 (64%), respectively, representing good explanatory power of the dependent constructs. The results in Table 8 revealed that all hypothetical relationships have a high effect size [104]. The Q^2^ value for OCE and platform preference was 0.36 and 0.42, respectively, indicating good endogenous constructs’ good predictive relevance.

**Table 8:** Significance of specific indirect effects

Indirect Path	β	T-value
PI → OCE → PFP	0.149*	2.51
SC → OCE → PFP	0.160*	2.71
Note: p < 0.05*, p < 0.001***		

#### Testing mediated effects

To test OCE's mediation, we followed the approach of Baron and Kenny [109]. The results specified that the direct effect of self-concept on platform preference was reduced by adding OCE as a mediator. Yet, the direct impact of platform interactivity on platform preference remained significant, giving rise to mediation on the relationship of platform interactivity and platform preference. Besides, OCE's subsequent effect on platform preference was also found significant, supporting mediation in our structural model. We also estimated mediation through the significance of indirect effects [110]. The results given in Table 9 indicate that OCE significantly mediates the relationships between platform interactivity and platform preference.

**Table 9:** Significance of total effects

Total Effects	β	T-value
PI → OCE → PFP	0.21**	2.61
SC → OCE → PFP	0.60***	7.76

*p* < 0.05*, *p* < 0.001***

Next, the degree of mediation is assessed by estimating the value of variance accounted for (VAF). The results in Table 10 suggested that OCE partially mediates the association between self-concept and platform preference. Subsequently, OCE partially mediates the association between platform interactivity and platform preference.

**Table 10:** Degree of mediation through VAF

H	Mediated paths	Indirect path I = (a*b*c)	Direct path (D = T-I)	Total effect(T)	VAF(I/T)	Results
H4a	PI → OCE → PFP	0.15	0.10	0.21	71%	Partial mediation
H4b	SC → OCE → PFP	0.16	0.72	0.60	26%	

#### Model fitness

The assessed GOF = 0.65 value proposes a good model fit, as given in Table 11. Lastly, the SRMR value of 0.078 indicates a good model fitness [104].

**Table 11:** Goodness of fit (GOF)

Constructs	AVE	R^2^
Platform interactivity	0.69	
Perceived value	0.66	
Perceived quality	0.74	
Self-brand image congruency	0.70	
Peer pressure	0.69	
Online consumer engagement	0.67	0.61
Platform preference	0.63	0.65
Average scores	0.68	0.63
AVE*R^2^	0.43	
GOF = $\surd$(AVE* R^2^)	0.65	

#### Importance performance map analysis (IPMA)

**Table 12:** Importance performance map analysis (IPMA)

Latent Constructs	Platform Preference	
	Importance	Performance
Platform interactivity	0.13	64.42
Self-concept	0.69	57.89
Online consumer engagement	0.15	58.94
Peer pressure	0.27	61.20

The values in bold direct the highest importance and highest performance

**Fig. 2:**
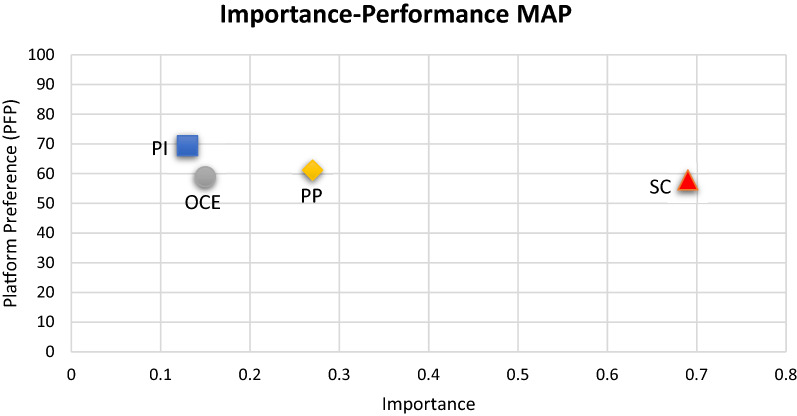
Importance performance map

The IPMA is an appreciated tool to evaluate the path coefficients practically and graphically. It has the potential to compare the importance and performance values of all exogenous constructs to predict the endogenous construct [104]. IPMA primary purpose is to recognize the precursor with better importance but the low performance and inversely [104]. The results are given in Table 12, and Fig. 2 shows that self-concept has a relatively low performance (57.89) but high importance (0.68) in predicting the platform preference. Likewise, platform interactivity has high performance (64.42) and low importance (0.13) in predicting the platform preference.

## Discussion and implications

### Discussion

As per our results, all hypotheses are accepted, which provided support to previous studies in a broader context related to the platform interactivity, self-concept, and OCE by proving the evidence of (H1) and (H2) acceptance [56, 73]. Further, it has reconfirmed that platform interactivity and self-concept are the critical determinants of OCE, ultimately leading to the adoption of platform preference. Among the antecedents for OCE, our findings indicate that “self-concept” is a relatively strong predictor of OCE and, therefore, positively impacts platform preference. Thus, retail managers are urged to concentrate on the means to build consumer engagement. The urge to socialize and buy on specific platforms that meet consumers' self-concept influences the platform choice behavior. However, different motives behind self-concept may have other driving power in consumer online engagement and choice. However, the TPB model elements, e.g., perceived quality, perceived value, and self-brand image congruency, have a uniform effect on the core construct of self-concept in our context. Further, the result of (H3) is also consistent with prior studies, as OCE is realized as a strong predictor of platform preference and purchase intention [79]. Furthermore, this study found that OCE's mediating role among platform interactivity, self-concept, and platform preference is consistent with previous empirical studies and matches these results [16]. This study acknowledged the partially mediated role of OCE among the said relationship, thus supporting H4a and H4b. Our findings empirically support the significance of OCE in making platform preferences and influence consumer purchasing decisions.

Lastly, China is described as a collectivist society, and people can be attributed to having a collectivistic approach in daily life. Our findings are persistent with the previous study and provide support to H5. Consistent with Muralidharan and Men [70], communication and engagement motivation are influenced by peer and social pressure in online shopping behavior in China. Social influence also has a significant effect on FDA adoption during the COVID-19 outbreak in China. These results also provided support to the work of Zhao and Bacao [7]. Our study results endorse that Chinese consumers are subject to peer pressure influencing OCE and platform preference. The results of this study conclude that consumer’s intention of using FDA during the COVID-19 epidemic not only suggestively influence platform preference but also intensely influenced by platform interactivity and self-concept, OCE, and social influence.

### Theoretical implications

This study has several avenues to add to the theoretical body of literature. This research attempts to empirically investigate test the antecedents that influence FDA user’s platform preferences in a novel way. We contribute to the existing literature of food buying selections by different FDA by integrating UGT by investigating OCE antecedents and platform preferences. The current study has incorporated the behavioral intention of FDA users and OCE factors such as platform interactivity and self-concept affecting platform preferences that can be utilized by the various researcher in other service industries, such as the online retail industry or online real estate segment. The unique findings predominantly highlight the importance and provide a framework based on UGT to understand better the strategic significance of FDA services considering the risks of COVID-19. Second, the current research enhances our understanding by integrating OCE's mediating role on the FDA among platform interactivity, self-concept, platform preference, a new phenomenon, and previously not been estimated. This research also outspreads the literature by classifying peer pressure plays a vital moderating role in the relationships between platform preferences and OCE in online foodservice business because peer pressure continuously affects our choices and social interaction. Our study emphasized that cultural values or context were essential factors affecting the magnitude of platform preference and OCE under peer pressure. Lastly, IPMA results designate all exogenous constructs' performance and importance in platform preference that offers in-depth insights.

### Managerial implications

The current study is early research that provides diverse and useful implications to managers and policymakers. Foodservice providers should concentrate on user preferences while using FDAs. This study increases the prevailing knowledge and benefits of FDAs, particularly in the context of the COVID-19 outbreak. This study has implications for enterprises like retailers operating mobile shopping apps, retailers selling their goods, and brands advertising/selling with those retailers. This study aims to provide marketers with different strategic tools to determine platform preferences for particular apps through empirical testing of key antecedents. This study supports FDA users and educates about various factors that marketers should focus on while making strategies for OCE and platform preference, thus encouraging potential customers to choose relevant food applications. The FDA has become progressively popular and useful platforms for the endurance of the foodservice business in a specific lockdown situation (COVID-19) and continuously developing after crises.

China is a culturally diverse country with an increasing number of foreign nationals from multiple races and nations. Moreover, it is also the world’s biggest country of active internet users. There is widely adopted a preference for mobile apps use in the community in every walk of daily lives, including food delivery business. Online food businesses should take emphasis on promoting the cognitive, social, and behavioral elements of OCE. Delivery app operators must streamline their value chain to support an accurate and timely flow of information, product, and services. To do so, vendors of delivery apps need to verify that the content they deliver is of the highest value in terms of reliability. To attain this purpose, retailers should continuously update their menus and considerable variation in prices to reduce user annoyance. They should also make consumers fully aware of their credibility through a detailed presentation of restaurant information. When choosing the FDA, consumers find themselves significantly influenced by their peers, suggesting that delivery service providers must be diligent in following word-of-mouth marketing. Mobile apps could soon become a retailer’s most vital sales platforms, mitigating the condition for immediate analysis of the customer’s perceptions. Because of the rapidly increasing online business model and the increasing usage of mobile internet applications, China's online food distribution has become a booming market. Thus, our research provides the infrastructure providers and restaurants with beneficial and valuable knowledge on making competitive business strategies for the foodservice industry in China.

### Limitation and future research

The authors confronted several limitations while conducting this research. The authors have primarily focused on OCE and platform preference by FDA users in the hotel industry. The framework may not be explicitly valid for other industries such as grocery and tourism services (such as Makemytrip.com, Yatra.com) and information (such as Tripadvisor.com, Yelp.com) often relies heavily on online evaluations for potential consumer adoptions. It is also suggested that this research’s boundaries are extended with diverse time horizon (during and post-COVID-19 lockdown) and regions with the end goal to have a more thorough examination. A longitudinal survey also helps to evaluate comprehensive results.

Further, cultural factors are ignored due to time and funding restrictions; however, future studies may thus examine relevant cross-cultural variations in the sense of channel preferences and their effect on behavioral intentions. Other potential moderating variables, such as discount rate and taste preference, could be included in future research. Therefore, more research may establish a different holistic approach by examining offline and online interactions to understand better how to provide a better food delivery service. Internet platforms also have different scopes, structures, cultures, and norms that can affect engagement [111].

### Conclusions

The current study investigated the effect of platform interactivity and self-concept on OCE and further on platform preference, specifically in FDA in the Chinese food sector during the COVID-19 epidemic. Our work also provides experiential evidence regarding the role of OCE as a mediator of the relationships among platform interactivity, self-concept, and platform preference.

## Data Availability

The datasets used and/or analyzed during the current study are available from the corresponding author on reasonable request.

## Abbreviations

OCE: Online consumer engagement
FDA: Food delivery applications
SEM: Structural equation modeling
PI: Peer pressure
UGT: Usage and gratification theory
